# The effect of unisensory and multisensory information on lexical decision and free recall in young and older adults

**DOI:** 10.1038/s41598-023-41791-1

**Published:** 2023-10-03

**Authors:** Christopher Atkin, Jemaine E. Stacey, Katherine L. Roberts, Harriet A. Allen, Helen Henshaw, Stephen P. Badham

**Affiliations:** 1 NTU Psychology, Nottingham Trent University, Nottingham, UK; 2 Hearing Sciences, Mental Health and Clinical Neurosciences, School of Medicine, University of Nottingham, Nottingham, UK; 3 National Institute for Health and Care Research (NIHR), Nottingham Biomedical Research Centre, Nottingham, UK; 4 School of Psychology, University of Nottingham, Nottingham, UK

**Keywords:** Human behaviour, Cognitive ageing, Long-term memory, Sensory processing

## Abstract

Studies using simple low-level stimuli show that multisensory stimuli lead to greater improvements in processing speed for older adults than young adults. However, there is insufficient evidence to explain how these benefits influence performance for more complex processes such as judgement and memory tasks. This study examined how presenting stimuli in multiple sensory modalities (audio–visual) instead of one (audio-only or visual-only) may help older adults to improve their memory and cognitive processing compared to young adults. Young and older adults completed lexical decision (real word vs. pseudoword judgement) and word recall tasks, either independently, or in combination (dual-task), with and without perceptual noise. Older adults were better able to remember words when encoding independently. In contrast, young adults were better able to remember words when encoding in combination with lexical decisions. Both young and older adults had better word recall in the audio–visual condition compared with the audio-only condition. The findings indicate significant age differences when dealing with multiple tasks during encoding. Crucially, there is no greater multisensory benefit for older adults compared to young adults in more complex processes, rather multisensory stimuli can be useful in enhancing cognitive performance for both young and older adults.

## Introduction

Our brains integrate multisensory information (e.g., audio and visual) on a moment-by-moment basis to successfully navigate and respond to the environment. Given the importance of multisensory information in day-to-day life, and a relationship between sensory processing deficits and age deficits^1^, researchers have started to concentrate on multisensory processing to address age deficits in cognition. The current article focuses on the potential for multisensory stimuli to alleviate age deficits in verbal judgement and memory tasks, by reducing cognitive resources devoted to perceptual processing.

An encouraging message emerged from early reviews of multisensory processing, with older adults benefiting more (i.e., decrease in the magnitude of age deficit in performance) from multisensory stimuli than did young adults (for reviews^2,3^, however, a recent review demonstrates age differences to be more variable^4^). For example, this benefit has been found in several studies using simple low-level stimuli such as judgements of temporal order^5^, speeded discrimination^6^ and detection^7^. In contrast, learning and memory paradigms have shown that both young and older adults benefit equally from multisensory stimuli. For instance, in problem solving^8^ and word recall^9^. This conflicts with two areas of research: First, leading ageing theory has proposed mechanisms linking low-level age deficits in processing speed to all aspects of age-related cognition including memory^10^, so improvements in speed via multisensory processing should translate to learning and memory by freeing up time spent on perception. Second, working memory has been proposed to operate with an amodal central storage component that integrates individual streams of sensory input^11^ into higher order areas of the brain such as the hippocampus^12^. Therefore, aiding such integration should translate to deeper learning and memory processes, particularly for older adults suffering from sensory deficits^13^ and deficits in cognitive resources and working memory^14^.

A remarkable feature of sensory research is that some patterns of age deficits in cognition can be reproduced in young adults by simulating perceptual deficits. This has led to several hypotheses exploring the relation between sensory and cognitive decline (for reviews^1,15,16^). For example, young adults can show reduced memory when words are presented with perceptual noise, even when the words were successfully identified during encoding^17^ and older adults are less able than young adults to identify an auditory target that is embedded in perceptual noise^18^. Moreover, it has been shown that load (e.g., perceptual noise or dual-task) has a considerable negative impact on older adults’ performance^16,19^. For example, when older adults are asked to perform a dual-task which increases cognitive load, then typically their performance is disproportionally poorer than that of young adults^20,21^. This indicates that perceptual processing, particularly for cases of increased cognitive load, may require cognitive resources that would be typically used for other processes such as memory encoding and consolidation. Similarly, age deficits in cognition can be reduced by perceptual support^22^. Therefore, given the high prevalence of age-related sensory deficits, facilitating perception through multisensory stimuli may be a particularly effective method to free up cognitive resources and alleviate older adults’ declines in memory and cognition.

Prior to the current study, there was scant research on age differences in intentional memory recall as a function of multisensory information and cognitive load. One study found that multisensory information improved intentional memory recall to a similar extent in young and older adults^9^. However, this study used pictures rather than written-text in both the visual and the audiovisual conditions. It is well established that verbal and nonverbal information is processed in different areas of the brain, and as such the use of picture-sound information could explain the lack of an increased multisensory benefit in performance for older adults relative to young. It may be that these two systems differ in their capacity to benefit from multisensory information. Some research has investigated the effect of cross-modal distractors when encoding and recalling unisensory information (e.g., Ref.^23^; see^4^ for review), but not tasks where participants can benefit from congruent multisensory information, as in this study. Research that has explored the effects of cognitve load on memory performance has only investigated unisensory information in both young and older adults (e.g., Refs.^18,19^). Because there is likely to be a complex interplay between age-related changes in unisensory perception, multisensory integration and cognitive capacity^1,4^, it is important to study these factors in combination. Therefore, our study is the first to investigate age, unisensory and multisensory information, and perceptual and cognitive load, in the context of intentional memory recall.

The aim of the present study was to establish if multisensory enhancement could alleviate age deficits in more complex processes such as judgement and memory tasks, and to understand how perceptual and cognitive load affect multisensory enhancement in young and older adults. A novel paradigm was developed that included three types of tasks: (i) a lexical decision task, where participants decide if unisensory (written, spoken) and multisensory words are real words or pseudowords. This task evaluates the speed at which young and older participants process lexical information in different modalities. (ii) A memory recall task, where participants recall unisensory (written, spoken) and multisensory words, to evaluate participants’ recall of lexical information in different modalities. (iii) A dual-task incorporating both lexical decisions and intentional memory encoding of unisensory and multisensory words, to evaluate the impact of additional cognitive load on both lexical decisions and recall. In all tasks, the words were presented in isolation and in visual/auditory noise, to assess the impact of perceptual load on cognitive performance.

Given that previous studies (e.g., Ref.^6^) have identified a multisensory benefit over unisensory stimuli using simple tasks and a decrease in the magnitude of age deficit in performance, we predict that multisensory stimuli will improve perception of the target words, leading to (1) faster lexical-decision responses with multisensory compared with unisensory stimuli, and (2) an increased multisensory benefit for older adults than young adults when completing the lexical-decision task. As a consequence of facilitating speed, we predict that (3) memory recall will be improved for multisensory compared with unisensory stimuli^9^, with (4) the multisensory benefit greater for older adults than young adults when completing the word recall task^10^. The greater multisensory benefit that is predicted for older adults over young adults for faster lexical decision and memory recall is based on Processing Speed theory^10^. According to this theory, multisensory stimuli should enhance speed which should free up more time for encoding. Freeing up time should disproportionately benefit older adults relative to young adults as speed reflects a key aspect of age-related deficits in cognition^10^.

Previous studies on perceptual load^17–19,23,24^ and cognitive load^20,21,24^ show that load reduces performance, thus we predict that additional perceptual load (perceptual noise) and cognitive load (dual-task) will make the task more difficult, leading to (5) worse lexical-decision speed and memory recall in the presence of noise and a dual-task, compared with performing the tasks independently and without noise. The multisensory stimuli are expected to improve perceptual processing (e.g., Refs.^4–6^), leading to (6) a reduction in these load-induced costs with multisensory compared with unisensory stimuli. Due to age-related perceptual and cognitive decline^1^, we predict that (7) load-induced costs will be greater for older adults than young adults. This prediction is supported by a range of evidence which shows that load reduces performance to a greater extent in older adults compared to young adults^18–21^. However, if multisensory stimuli reduce the impact of age-related perceptual and cognitive decline^6^, we predict that (8) age differences in load-induced costs will be reduced for multisensory stimuli compared with unisensory stimuli (e.g., Refs.^4–6^).

Overall, most previous studies have investigated multisensory processing using simple tasks show that multisensory stimuli lead to greater improvements in processing speed for older adults than young adults. However, multisensory processing has not being investigated extensively using more complex tasks. The current study aimed to establish if multisensory enhancement can alleviate age deficits in more complex processes such as verbal judgement and memory encoding, as suggested by theory and the aging literature.

## Methods

### Study overview

Older (65 to 78) and young (19 to 30) adults completed lexical decision (words vs pseudowords) and word recall tasks, either independently (lexical decision task and encoding task) or in combination (dual-task; cognitive load), with and without perceptual noise (perceptual load). Together the three tasks investigated the effects of age (older, young), presentation modality (auditory, visual, audio–visual) and load (no load; LD + encoding; and LD + encoding + noise) on lexical decision task speed and free recall of real words. Task order (dual-task, lexical decision, encoding) and condition order (e.g., auditory, visual, audio–visual, auditory noise, visual noise, audio–visual noise) within task were counterbalanced using a Balanced Latin Squared Design.

#### Participants

A total of 31 young adults (21 Female) aged 19 to 30 years (*M* = 20.77, *SD* = 2.68) and 31 older adults (20 Female) aged 65 to 78 years (*M* = 70.65, *SD* = 4.06) took part in the experiment. A post-hoc power analysis was conducted to evaluate our sample size using MorePower 6.0.4^25^ for a mixed ANOVA design with one between measures factor with two-levels and two repeated measures factors, each with three-levels. The post-hoc analysis aimed to detect significant main effects, two-way, and three-way interactive effects with an alpha (α) of 0.05 and a medium effect size (η_p_^2^ = 0.06). Results indicated that a total sample of 62 participants in the current study to have a 0.89% power. The sample was collected at Nottingham Trent University using the University’s participant research panels. All participants reported normal or corrected to normal vision and were excluded if they reported any known memory impairments, dementia, impaired cognitive function and learning difficulties. Informed consent was obtained from all participants. Each participant received a £10 gift voucher for completing the 75-min study. Data collection was approved by Nottingham Trent University’s Business, Law and Social Sciences Research Ethics Committee and experiments were performed in accordance with the agreed guidelines and regulations.

Young adults (YA) and older adults (OA) did not differ significantly in their level of education, *t* < 1 (*M*_YA_ = 2.96, *SD*_YA_ = 0.74; *M*_OA_ = 2.77, *SD*_OA_ = 1.15). To assess cognitive functioning and hearing ability, participants completed the Digit Symbol Substitution test from the Wechsler Adult Intelligence Scale—Revised^26^ as a measure of processing speed, the multiple-choice part of the Mill Hill vocabulary test^27^ as a measure of crystallized intelligence, and the Speech in Noise test^28^ as a measure of hearing. The results were consistent with the literature^29,30^: YA performed better than OA at the speed task, *t*(60) = 4.27, *p* < 0.001 (*M*_YA_ = 63.06, *SD*_YA_ = 10.14; *M*_OA_ = 52.52, *SD*_OA_ = 9.29), and OA performed better than YA at the vocabulary task, *t*(60) = 6.83, *p* < 0.001 (*M*_YA_ = 15.84, *SD*_YA_ = 3.68; *M*_OA_ = 22.42, *SD*_OA_ = 3.91), and YA performed better than OA at the listening task *t*(60) = 4.91, *p* < 0.001 (*M*_YA_ =  − 11.36, *SD*_YA_ = 2.56; *M*_OA_ =  − 8.18, *SD*_OA_ = 2.55). All participants self-reported their hearing ability to be better than or equal to fair, and their hearing performance was better than or equal to 0 dB signal noise ratio as measured by the Speech in Noise task^28^. In addition, a verbal confirmation of clarity and ease of hearing the stimuli was obtained from all participants in a sound check during participation.

#### Design and procedure

The conceptual differences between perceptual and cognitive load are widely discussed in the literature and clarification on the precise definitions of these terms are still under debate^24^. However, we take the perspective that it is inherently difficult to manipulate *pure* perceptual or cognitive load. For example, in auditory tasks, it can be challenging to increase perceptual load without also increasing the cognitive demands associated with decoding the auditory scene^31^. Similarly, to raise perceptual load without increasing some cognitive burden is also challenging^31–34^. Taking this into account, for the purpose of the current study, we state that our manipulations are predominantly perceptual and predominantly cognitive load, rather than pure manipulations of perceptual and cognitive load. In terms of providing a definition for each of these terms, we define perceptual load as a modification to the signal that predominantly compromises its integrity, such as background speech or visual noise that is overlaid. In comparison, cognitive load is defined as load that predominantly impacts the recruitment of central processing resources due to concurrent processing (such as a dual-task), rather than a signal distortion.

### Dual-task (lexical decision and encoding)

**Figure 1:**
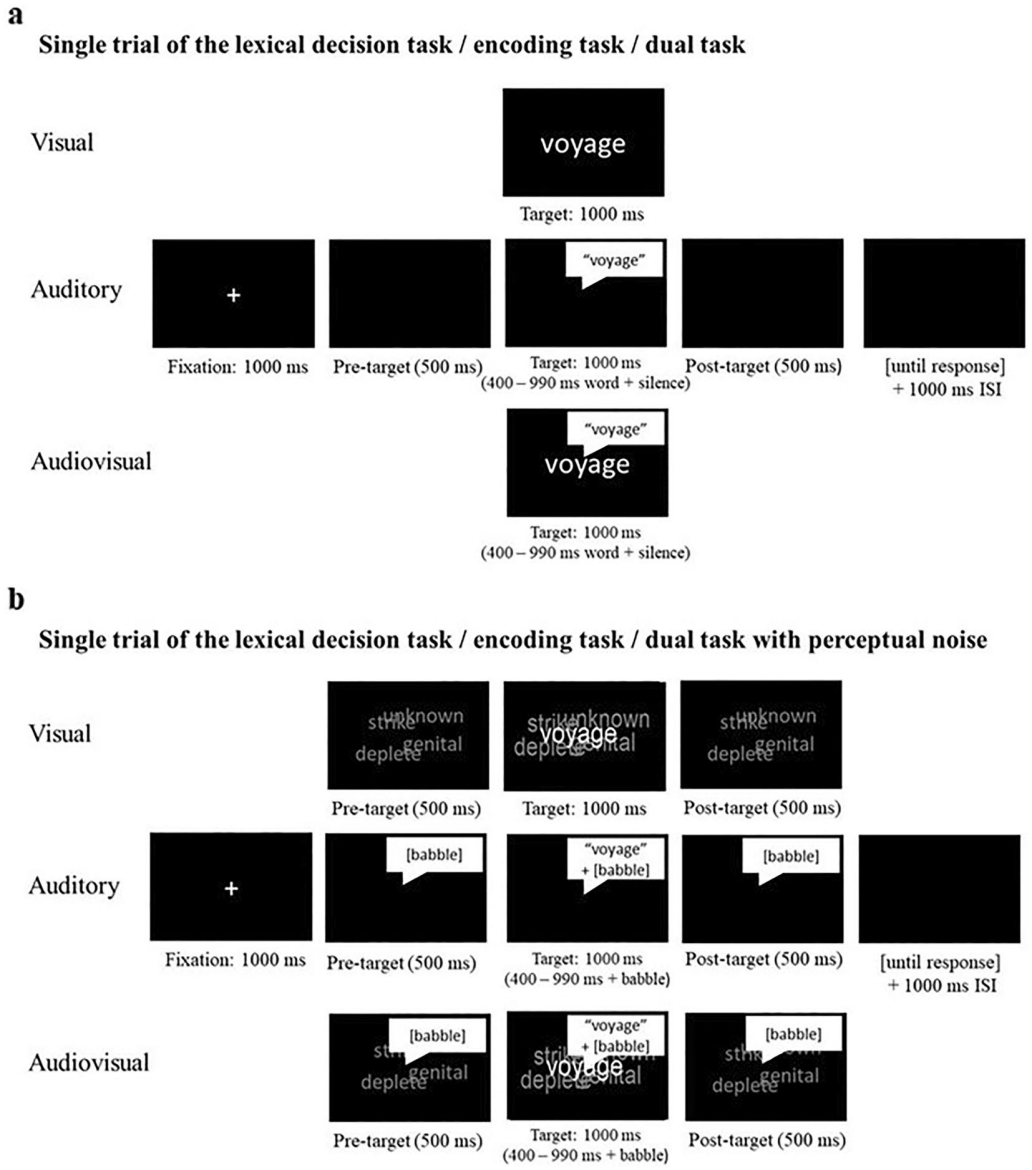
(**a**) Single trial of the lexical decision task (LD)/encoding task/dual-task (lexical decision and encoding) for each modality (audio, visual, audio–visual). In the LD task participants made a decision about real words and pseudowords. In the encoding task, participants were presented with real words and later asked to recall them. The dual-task included the lexical decision task followed by free recall of the real words only. (**b**) Single trial of the LD/encoding task/dual-task with perceptual noise. Procedure is the same as explained above with added perceptual noise in each modality: visual modality contained visual babble on screen, audio modality contained audio babble, and audio–visual modality contained both audio and visual babble.

In the dual-task, young and older adults had to decide between words and pseudowords (lexical decision) and remember only the words for a later memory test. Words and pseudowords were presented auditorily, visually or audio–visually. Participants completed the task with no noise (silence) and with noise (audio noise only, visual noise only or both audio and visual noise)—see Fig. 1.

Six conditions were created by combining three levels of presentation modality (auditory, visual, audio–visual with 2 levels of noise (no noise, noise). Participants completed 144 trials across the six conditions (auditory, visual, audio–visual, auditory noise, visual noise, audio–visual noise). Separate word lists were created for the 6 conditions, each containing 12 words and 12 pseudowords. Word lists were randomised such that they could be assigned to any of the six conditions.

Each trial began with a white fixation cross, presented at the centre of the display on a black background for 1000-ms. After which, a 500-ms blank black screen was presented during visual, auditory, and audio–visual trials. Instead of a blank screen, the visual and audio noise trials contained visual or auditory noise for 500-ms, respectively, and the audio–visual noise trials contained both visual and auditory noise for 500-ms.

The target stimulus (word or pseudoword) was presented in the centre of the screen for 1000-ms, while the screen remained blank for auditory trials. The duration of the auditory target stimulus for auditory and audio–visual trials varied between 400 and 990-ms depending upon the duration of the spoken target word. Silence (or babble in the noise conditions) were added to each of the audio and audio–visual trials so that each trial was presented for 1000-ms. The visual noise continued in the visual and audio–visual noise trials for the duration of the visually presented target stimulus (1000-ms).

After the target stimulus was presented in the no-noise conditions (visual, auditory, and audio–visual) a 500-ms blank screen was presented. In contrast, in the noise conditions the babble noise continued in the auditory and audio–visual noise trials for 500-ms after the offset of the target stimulus. Similarly, the visual noise continued in the visual and audio–visual noise trials for 500-ms after the offset of the target stimulus. On audio–visual trials, visual and auditory onsets were simultaneous, and the text was always congruent with the auditorily presented stimulus. Finally, a blank screen was presented, and participants indicated whether the target stimulus was a word or pseudoword by pressing the ‘m’ or ‘z’ keys, respectively, and were instructed to respond as quickly as possible while maintaining accuracy. Trials were separated with an inter-stimulus interval of 1000-ms.

Following the final trial, participants completed a short distractor task in the delay (backwards counting aloud from 300 in 3 s) for 30 s. After which, participants completed a free recall memory task by vocalising the 12 possible words (not the pseudowords) that were presented in the lexical decision task, which were written down by the experimenter. Participants has 60 s to recall as many of the words as possible.

### Encoding task

This task was similar to the dual-task (presented above), but did not include noise factors, or the lexical decision element of the task. Instead, young and older adults were presented with one of 3 sets of 12 words with each set containing only audio, only visual or audio–visual words (see Fig. 1). Participants were instructed to remember the presented words for a later memory test.

As in the dual-task, the three conditions were auditory, visual, and audio–visual. At the end of each condition, participants completed a short distractor delay (backwards counting from 300 in 3 s) before completing a free recall memory test. Participants completed 36 trials across the three conditions (auditory, visual, audio–visual). Three comparable word lists were created for the three conditions, each containing 12 words. Free recall followed each of the three lists. Word lists were randomised such that they could be assigned to any of the three conditions.

### Lexical decision task

This task was similar to the dual-task (presented above) but did not include added noise, the delay task, or the free recall element of the dual-task. Instead, young and older adults had to decide between words and pseudowords (classic lexical decision task) which were presented auditorily, visually or audio–visually (see Fig. 1).

The three conditions were identical to those in the dual-task (auditory, visual, and audio–visual). Participants completed 72 trials across the three conditions (auditory, visual, audio–visual). Each condition was allocated 24 (12 words, 12 pseudowords) words. 3-word lists were created for the three conditions, each containing 12 words and 12 pseudowords. Word lists were randomised such that they could be assigned to any of the three conditions.

## Materials and apparatus

### Equipment

All tasks were run on a Lenovo ThinkCentre M79 10J7 using a 27″ monitor (60-Hz refresh rate) with a 2560 × 1440-pixel resolution. Sounds were presented at ~ 72 dB SPL via two front facing speakers (Logitech X-140 S-0264B) and calibrated by presenting the stimuli over the speakers and measured using a microphone (ACO 7052E) connected to a sound level meter (SVAN 977). The dual, lexical decision and encoding tasks were programmed using Psychopy 1.1.3^35^. Viewing distance was set at ~ 57 cm for each task.

### Stimuli

Words and pseudowords (target words) were selected from the Auditory English Lexicon Project^36^ and included the corresponding sound files. All words were nouns and were controlled for word length (4–6), word frequency (occurrences in the English language, per million words, 2.5–3.5), number of phonemes (4–7), number of syllables (2–3) and age of acquisition (4–10). Pseudowords were controlled for word length (4–6), number of phonemes (4–7) and number of syllables (2–3). Target word property statistics were not significantly different (*p* > 0.090) across word lists. The target words were spoken by a British male (M3^36^) for the auditory conditions and were presented on a blank screen. For visual conditions, the target words were presented in Arial font, white (RGB: 1, 1, 1) text, 60 pixels (visual angle 1.36°) and were presented on the centre [X, Y (0, 0)] of a black screen.

### Noise stimuli

Auditory noise stimuli were created using an existing database^37^. The database consists of a series of individual talkers that describe a previously viewed video or cartoon strip. Each recording is 30-s long and describes the same scene. Two separate 30-s male talker recordings were used to create two-talker babble^37^. Audacity software was used to remove any silences and expressions (e.g., “erm”, “um”). On each auditory or audio–visual noise trial, 2-s of segments were randomly sampled from the two-talker babble. The signal-to-noise ratio (SNR) was presented at + 12 dB. The two-talker babble was mixed with the target stimuli, and both came from the same speaker.

Visual noise stimuli were created using previous research^38^. The visual babble stimuli consisted of four overlapping grey (RGB: 0, 0, 0) Ariel font words (55 pixels, visual angle 1.25°) which were presented on a black screen. The words jitter randomly on the X axis between − 75 and − 60 pixels, left of centre and between 60 and 75 pixels, right of centre. On the Y axis, the words will be placed in four random positions, 20 or 30 pixels above centre and − 30 or − 20 below centre. An independent reviewer inspected the visual babble stimuli and identified 0.52% of the words as ‘taboo’ or arousing.

## Results

### Data preparation and statistical analysis

Data were analysed in accordance with the respective pre-registration (10.17605/OSF.IO/3QND6). All analyses were performed using R^39^. Response time data were trimmed to exclude responses that were too fast (< 100 ms) or too slow (> 4000 ms). Data trimming limits were deemed acceptable to eliminate trials in which participants might not have been attending to the task sufficiently. Trimming resulted in the removal of 14 (0.10%) trials for young adults and 20 (0.15%) trials for older adults.

For the free recall data, a 2 × 3 × 3 mixed ANOVA (age: young, older adults × modality: auditory, visual, audio–visual × load: lexical decision only, lexical decision + encoding, lexical decision + encoding + noise), was conducted on proportion of list recalled. For the lexical decision data, a 2 × 3 × 3 mixed ANOVA (age: young, older adults × modality: auditory, visual, audio–visual × load: lexical decision only, lexical decision + encoding, lexical decision + encoding + noise), was conducted on log reaction times (cf.^40^) and then separately on accuracy (the accuracy data showed ceiling effects and is less informative. It is shown in the Supplementary Material). Post hoc analyses using Bonferroni corrections were used to investigate significant interactions. Greenhouse–Geisser correction was applied for non-sphericity. Effect sizes for ANOVAs are reported using partial eta squared.

### Free recall analysis

The means and standard deviations for proportion of words successfully recalled can be seen in Table 1. A summary of ANOVA effects for proportion of words successfully recalled can be found in Table 2. ANOVAs for serial position effect and mean number of intrusions can be found in Supplementary Material. As expected, there was a significant effect of age with young adults remembering more words than older adults. There was a significant effect of modality, with both groups recalling more words when information was presented audio–visually compared to auditorily (Hypotheses 3), but no interaction between age group and modality, showing no additional multisensory benefit for older adults (Hypothesis 4).

**Table 1:** Proportion of words successfully recalled in the free recall task and log 10 response times ms (RTs) on the lexical decision task, for each modality (audio, visual, audio–visual), age group (young, older) and load condition (encoding only, LD + encoding, LD + encoding + noise). Standard deviations are shown in parentheses.

	Audio		Visual		audio–visual	
	Young	Older	Young	Older	Young	Older
Recall						
Encoding only	0.41 (0.18)	0.27 (0.15)	0.41 (0.21)	0.26 (0.19)	0.41 (0.23)	0.31 (0.15)
LD + encoding	0.47 (0.18)	0.21 (0.12)	0.47 (0.19)	0.26 (0.13)	0.49 (0.20)	0.22 (0.12)
LD + encoding + noise	0.39 (0.19)	0.19 (0.13)	0.48 (0.20)	0.21 (0.11)	0.46 (0.22)	0.27 (0.17)
Log RTs						
LD only	3.05 (0.07)	3.11 (0.08)	2.84 (0.07)	2.92 (0.09)	2.88 (0.08)	2.95 (0.08)
LD + encoding	3.08 (0.08)	3.11 (0.08)	2.92 (0.09)	2.95 (0.08)	2.95 (0.08)	2.99 (0.09)
LD + encoding + noise	3.07 (0.08)	3.12 (0.09)	2.92 (0.09)	2.97 (0.09)	2.94 (0.10)	3.00 (0.10)

**Table 2:** Summary of ANOVA effects for memory recall and lexical decision log 10 response time (RT) data.

	*F*	DF	*p*	η_p_^2^	Post-hoc tests^a^
Recall					
Modality	4.55	2, 120	= 0.013	0.07	AV > A
Age	36.49	1, 60	< 0.001	0.38	Y > O
Load	4.20	1.61, 96.71	= 0.299	0.02	
Modality × age	< 1				
Age × load	7.90	2, 120	< 0.001	0.12	Older_E_ > Older_LD+E+N_, Young_LD+E_ > Young_E_|in all load conditions: Y > O (Fig. 2)
Modality × load	1.72	4, 240	= 0.147	0.03	
Modality × age × load	1.91	4, 240	= 0.110	0.03	
LD RT					
Modality	510.67	1.64, 98.39	< 0.001	0.90	V > AV and A, AV > A
Age	8.87	1,60	= 0.004	0.13	O > Y
Load	29.40	1.82, 109.33	< 0.001	0.33	LD > LD + E and LD + E + N
Modality × age	< 1				
Age × load	4.88	2, 120	= 0.009	0.08	Young_LD_ > Older_LD_, Young_LD+E+N_ > Older_LD+E+N_|Older_LD_ > Older_LD+E_ and Older_LD+E+N,_ Young_LD_ > Young_LD+E_ and Young_LD+E+N_ (Fig. 3a)
Modality × load	7.06	4, 240	< 0.001	0.11	AV_LD_ > AV_LD+E_ and AV_LD+E+N_, V_LD_ > V_LD+E_, and V_LD+E+N_|V_LD_ > A_LD_ and AV_LD_, AV_LD_ > A_LD,_ V_LD+E_ > A_LD+E_ and AV_LD+E,_ AV_LD+E_ > A_LD+E_, AV_LD+E_ and V_LD+E_ > A_LD+E_ (Fig. 3b)
Modality × age × load	< 1				

For post-hoc tests: > faster response time/better recall, < slower response time/poorer recall.

*A* audio, *V* visual, *AV* audio–visual, *O* older adults, *Y* young adults, *LD* lexical decision only, *E* encoding only, *LD* + *E* lexical decision plus encoding, *LD* + *E* + *N* lexical decision plus encoding plus noise.

^a^Significant results are only reported.

**Figure 2:**
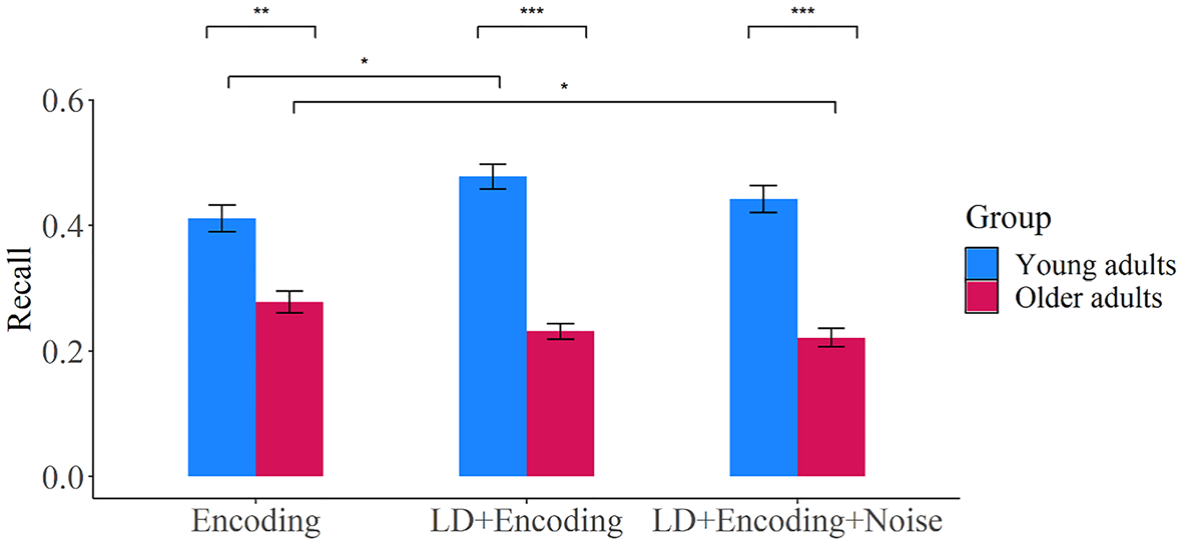
Recall (proportion correct) for age (older and young) by load (encoding only, LD + encoding and LD + encoding + noise). Error bars indicate standard error. *< 0.05, **< 0.01, ***< 0.001.

There was no main effect of load (Hypothesis 5), and no two-way interaction between modality and load (Hypothesis 6). There was a significant two-way interaction between load and age (Hypothesis 7; Fig. 2). Pairwise comparisons, detailed in Table 2, showed that older adults had better recall in the encoding-only condition compared to the dual-task plus noise condition, whereas young adults had better recall in the dual-task compared to the encoding-only condition. There was no three-way interaction between modality, load, and age (Hypothesis 8), indicating that multisensory stimuli did not influence the interaction between load and age.

### Lexical decision analysis

The means and standard deviations for log response times can be seen in Table 1. A summary of ANOVA effects for log response times can be found in Table 2. Young adults had faster response times than older adults.

Stimulus modality had a significant effect on RTs (Hypothesis 1). Responses to audio–visual stimuli were faster than responses to auditory-only stimuli, but slower than responses to visual-only stimuli.

We predicted that older adults would have a greater multisensory benefit than young adults when completing the lexical decision task (Hypothesis 2), but there was no interaction between age and modality. There was therefore no evidence of an increased multisensory benefit for older adults.

Additional perceptual and cognitive load had a negative effect on lexical-decision responses (Hypothesis 5), with participants responding faster in the lexical-decision only task than in the dual-task and dual-task plus noise conditions.

**Figure 3:**
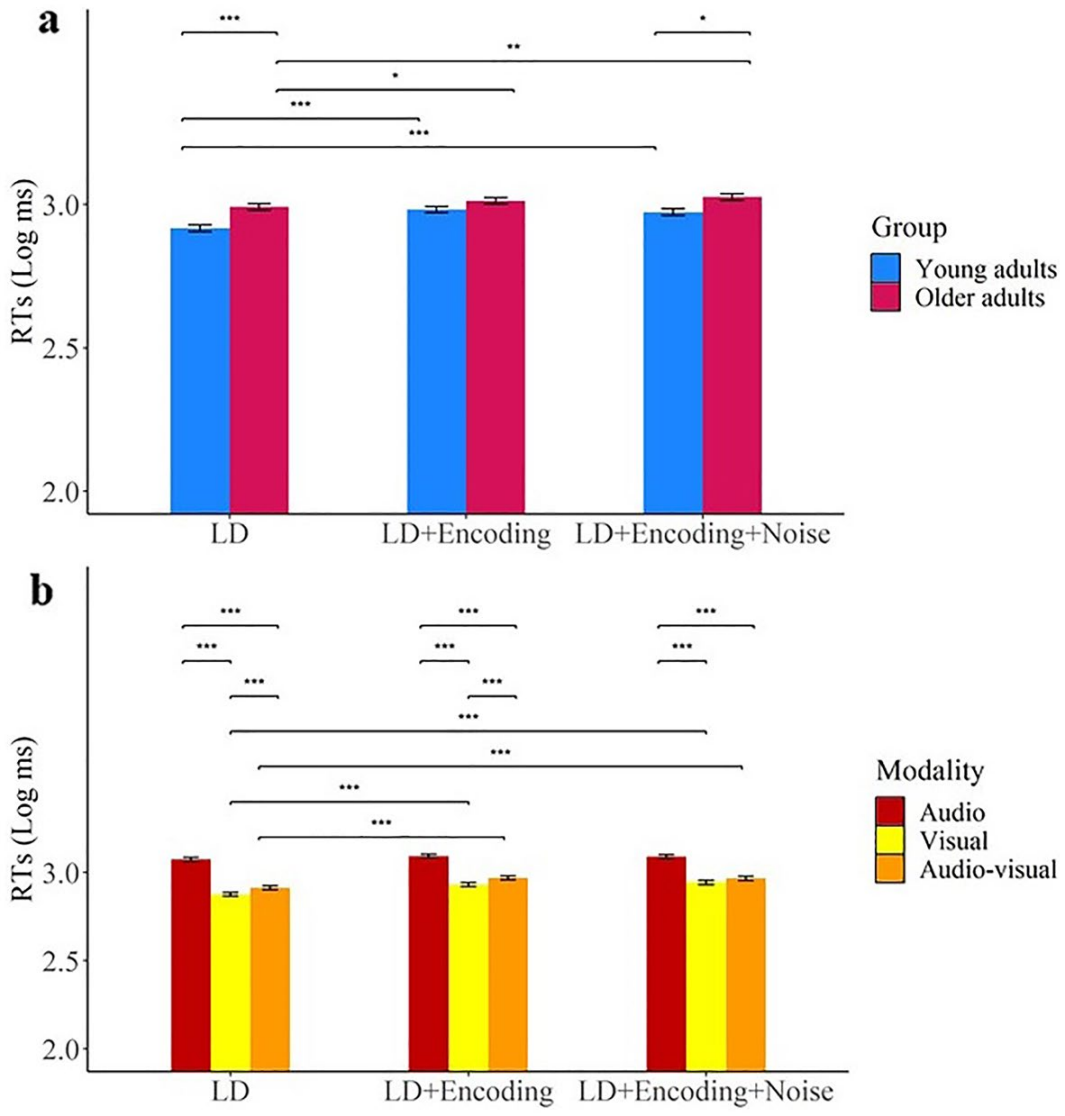
(**a**) Lexical decision RTs (Log 10 response time, ms) for age (older and young) by load (LD only, LD + encoding and LD + encoding + noise). (**b**) Lexical decision RTs (Log 10 response time, ms) for modality (audio, visual and audio–visual) by load (LD only, LD + encoding and LD + encoding + noise). Error bars indicate standard error. *< 0.05, **< 0.01, ***< 0.001.

We predicted that multisensory stimuli would reduce the negative impact of increased load (Hypothesis 6). There was a two-way interaction between modality and load in response times (Fig. 3b). Pairwise comparisons (Table 2), showed that in the visual and audio–visual conditions, responses were faster in the lexical-decision only condition than in the dual-task and dual-task plus noise conditions, whereas for the audio-only condition there was no effect of load on response times (although this modality by load interaction effect for response times becomes non-significant when speech in noise ability is entered as a covariate).

We predicted that increased load would have more impact on older participants than young participants (Hypothesis 7). There was a significant interaction between age and load (Fig. 3a). Pairwise comparisons (Table 2) show that young adults had faster responses than older adults when the lexical-decision task was completed on its own and when completed with both encoding and noise, but young and older participants had similar response times when the lexical decision task was completed with the encoding task but without noise. There was no three-way interaction between age, modality, and load, indicating that multisensory stimuli did not moderate the interaction between age and load.

## Discussion

This study was designed to determine if multisensory enhancement could alleviate age deficits in more complex processes such as verbal judgement and memory tasks, and whether multisensory enhancement reduces any costs associated with increased cognitive and perceptual load for older compared to young adults. We developed a novel linguistic paradigm that measured memory recall and lexical decision reaction times under different levels of perceptual and cognitive load. As expected, older adults had worse recall than young adults, along with slower lexical decisions. Both young and older adults had improved recall of audio–visual stimuli compared with auditory-only and made faster lexical decisions for audio–visual stimuli than auditory-only stimuli (but not faster than visual-only stimuli), showing multisensory enhancement. However, there was no evidence of a greater multisensory benefit for older adults over young adults, demonstrating that the disproportional multisensory enhancements that older adults have previously shown relative to young adults on perceptual decision tasks (e.g., Refs.^6,7^) do not transfer to more complex cognitive tasks. Interestingly, young adults were better able to remember words when performing lexical decisions and encoding, compared with encoding alone, while older adults’ recall was reduced when performing lexical decisions and encoding in conjunction with perceptual noise. Our findings provide evidence that age disparities exist when performing multiple tasks during encoding and multisensory stimuli can be helpful in specific circumstances for increasing complex cognitive performance for both age groups.

### Free recall

With regards to memory performance, it was expected that audio–visual information would speed-up processing and reduce the effects of cognitive load during encoding and improve memory recall to a greater degree for older than for young adults. Older adults’ memory was poorer than young adults which is aligned with the aging literature^41^. The findings indicate an overall memory benefit for audio–visual information over audio only information, however this was similar for both young and older adults. Older adults’ memory was reduced when completing the dual-task with perceptual noise compared to completing the encoding-only task, which is consistent with previous literature on dual-task performance^20^ and perceptual noise^16,18,19^. One possible explanation is that older adults are less able to inhibit task-irrelevant distractors. Indeed, according to *Attentional Load Theory*, an increase in cognitive load enhances the processing of irrelevant distractors^42^. Moreover, older adults are less able to accurately weigh relevant and irrelevant sensory influences from both the internal and external environment^2^. Instead, older adults process all available sensory information, even when it is detrimental to performance^43^. Another explanation is that during language encoding, older adults rely more heavily on top-down strategies, as compared to bottom-up, sensory encoding strategies, which are commonly impaired in ageing^44,45^. Finally, given that older adults have fewer processing resources, they may spend a large portion of those resources identifying the stimulus word in noise, leaving insufficient resources for processing the item in memory^19^.

In contrast, young adults’ memory recall improved in the dual-task. Evidence has regularly found that young adults’ performance deteriorates when performing a dual-task^20^. However, our unusual finding may be consistent with the *Attentional Boost Effect* (for review see^46^), which occurs under divided attention encoding and can result in improved memory for young adults^47^. Moreover, it has been found that performing a dual-task may result in a substantial improvement in the sensory processing of target words during the encoding phase in young adults^48^.

### Lexical decision

Evidence has shown that older adults gain a speed advantage over young adults when presented with multisensory information^6,7^. Often this advantage is assumed to be due to declines in unisensory processing with multisensory information acting as a compensatory mechanism to alleviate these age-related deficits (e.g., Ref.^49^; however, see^2^ for discussion). Interestingly, the current study did not find a multisensory response-time benefit for older adults. Rather, a visual-only benefit was found over audio–visual information for both young and older adults. One possible explanation for the lack of multisensory benefit in older adults compared to young here, could be due to the type of task and stimuli used^4^. In the current study, participants had to perform a lexical decision task that requires lexical access and processing of words and pseudowords, whereas other studies typically use a simple visual colour decision task (e.g., red/blue disks^6^). It could be that the multisensory benefit is only found in older adults when using simple coloured visual stimuli which do not require greater cognitive processing (e.g., lexical access and processing).

The lack of response-time benefit for audio–visual information over visual only information is also relevant to The *Processing Speed Theory*^10^. According to this theory, mechanisms connecting low-level deficits in processing speed propagate to all aspects of cognition. As such, improvements in speed via multisensory processing should translate to learning and memory and should therefore benefit older adults to a greater degree than young adults. However, we found no evidence to suggest that older adults gain a greater multisensory benefit in lexical decision judgement or memory recall. Rather the results show similar effects for young and older adults. Taken together, these findings support the idea that higher order cognitive processes work independently of an age-related multisensory enhancement^3^.

The visual-only response-time benefit found over audio–visual information extends the literature investigating lexical decisions and sensory information. For example, Zunini et al. investigated lexical processing for audio only, visual only and audio–visual information in young adults and found a response-time benefit when processing visual information compared to audio–visual^50^. However, Zunini et al.’s sample only included young adults^50^. In the current study, both young and older adults were tested, and the effect was found to be independent of age. An explanation for the visual benefit over audio–visual information is that the visual input provides the quickest path to the lexicon, and the audio portion of the audio–visual information provides a lag in processing of this information.

It is possible that the perceptual load (auditory babble and visual babble) used in the current study may have induced some cognitive interference due to the demands of the babble stimuli (i.e., two-talker spoken words and overlaid written words). However, auditory and visual babble stimuli have been used in previous research and is a well-used operational method for investigating perceptual load (e.g., Ref.^38^). Furthermore, we take the perspective that perceptual and cognitive load rely partly on the same pool of resources (e.g., Refs.^32–34^, also see^24^ for discussion on load theory), a pool which through experimental design can be assigned more heavily to either perceptual or cognitive processing. Nonetheless, future research should aim to create purer manipulations of perceptual and cognitive load to further investigate whether multisensory information can reduce costs associated with aging and load.

In summary, the present findings show that older adults had worse recall than young adults, along with slower lexical decisions. Older adults were better able to remember words when encoding independently. In contrast, young adults were better able to remember words when encoding in in combination with lexical decisions. Both young and older adults had improved recall of audio–visual stimuli compared with auditory-only and made faster lexical decisions for audio–visual stimuli than auditory-only stimuli, showing multisensory enhancement in both recall and lexical decisions. Finally, we concluded that the disproportional multisensory benefits that older adults have shown in previous studies (e.g., Refs.^6,7^) do not transfer to more complex cognitive tasks, indicating a more complete relationships between cognition and perception in ageing and that the multisensory benefit does not propagate fully from perceptual to cognitive tasks. Nevertheless, these findings show that age differences increase when performing dual-tasks, and multisensory stimuli can be useful in enhancing cognitive performance for all age groups.

### Supplementary Information

Supplementary Information.

## Data Availability

The datasets analysed during the current study are available in the Open Science Framework repository, 10.17605/OSF.IO/3QND6.
