# Supplementary material for: The effect of unisensory and multisensory information on lexical decision and free recall in young and older adults
Source: Sci Rep. 2023 Oct 3;13:16575. doi: 10.1038/s41598-023-41791-1 (PMC10547689; doi:10.1038/s41598-023-41791-1)
Supplement: Supplementary file 1 — Supplementary Information. [file 41598_2023_41791_MOESM1_ESM.docx]

**The effect of unisensory and multisensory information on lexical decision and free recall in young and older adults**

Christopher Atkin, Jemaine E Stacey, Katherine L Roberts, Harriet A Allen, Helen Henshaw, Stephen P Badham

**Supplementary material**

**Lexical decision accuracy data**

A 2 (age: young, older adults) x 3 (modality: auditory, visual, audio-visual) x 3 (load: lexical decision only, lexical decision + encoding, lexical decision + encoding + noise) mixed ANOVA was conducted on accuracy.

The means and standard deviations for accuracy can be seen in Supplementary Table 1. A summary of ANOVA effects for log response times can be found in Supplementary Table 2.

**Table S1.** Proportion correct (accuracy) on the lexical decision task, for each modality (audio, visual, audio-visual), age group (young, older) and load condition (encoding only, LD + encoding, LD + encoding + noise). Standard deviations are shown in parentheses.

|  | Audio | | Visual | | Audio-visual | |
| --- | --- | --- | --- | --- | --- | --- |
|  | Young | Older | Young | Older | Young | Older |
| **Accuracy** |  |  |  |  |  |  |
| LD only | .95 (.05) | .96 (.04) | .95 (.05) | .98 (.03) | .98 (.03) | 1.0 (.02) |
| LD + encoding | .96 (.05) | .97 (.04) | .96 (.06) | .99 (.03) | .98 (.03) | 1.0 (.01) |
| LD + encoding + noise | .93 (.06) | .93 (.06) | .95 (.07) | .99 (.02) | .99 (.03) | .99 (.02) |

**Table S2.** Summary of ANOVA effects for Lexical decision (LD) accuracy data.

|  | ***F*** | **DF** | ***p*** | **η_p_^2^** |  | **Post-Hoc Tests^a^** |
| --- | --- | --- | --- | --- | --- | --- |
| **LD accuracy** |  |  |  |  |  |  |
| Modality | 42.48 | 1.68, 104.15 | < .001 | .42 |  | AV > V & A , V > A |
| Age | 13.12 | 1, 60 | < .001 | .18 |  | O > Y |
| Load | 4.94 | 2, 120 | = .009 | .08 |  | LD+E > LD+E+N |
| Modality*Age | 3.68 | 2, 120 | = .028 | .06 |  | AV_older_ > AV_young,_ V_older_  > V_young_ \| AV_older_ > A_older_ , V_older_ > A_older,_ AV_young_ > A_young_ &V_young_ (Figure S1) |
| Age * Load | < 1 |  |  |  |  |  |
| Modality * Load | 5.21 | 3.14, 188.34 | = .003 | .07 |  | A_LD_ & A_LD+E_ > A_LD+E+N_ \| AV_LD_ > V_LD_ & A_LD_, AV_LD+E_ > A_LD+E_, AV_LD+E+N_ > V_LD+E+N_ & A_LD+E+N_, V_LD+E+N_ > A_LD+E+N_ (Figure S2) |
| Modality*Age*Load | < 1 |  |  |  |  |  |

Notes. A = Audio, V = Visual, AV = Audio-visual. O = older adults, Y = young adults. LD = Lexical Decision only E = Encoding only, LD+E = Lexical Decision plus encoding, LD+E+N = Lexical Decision plus encoding plus noise. For post-hoc tests: > better accuracy, < poorer accuracy. ^a^ significant results are only reported.

Older adults were more accurate than young adults. Stimulus modality had a significant effect on accuracy. Responses were more accurate to audio-visual stimuli than auditory-only stimuli. Audio-visual stimuli were responded to more accurately than visual-only stimuli.

There was an interaction between age and modality for lexical-decision accuracy (Figure S1). Table S2 details the pairwise comparisons, which show that while both young and older adults had higher accuracy in the audio-visual condition compared with the audio-only condition, only young adults showed an accuracy benefit for audio-visual stimuli compared with visual stimuli. There was therefore no evidence of an increased multisensory benefit for older adults.

Additional perceptual and cognitive load did have a negative effect on lexical-decision responses, with participants responding more accurately in the dual task condition than the dual-task plus noise condition. There was no significant interaction between age and load for accuracy.

There was a two-way interaction between modality and load for accuracy (Figure S2). Pairwise comparisons (Table S2), showed that accuracy varied as a function of load in the audio-only condition, where noise reduced accuracy relative to the other two conditions, but there was no effect of load on accuracy in the visual and audio-visual conditions.


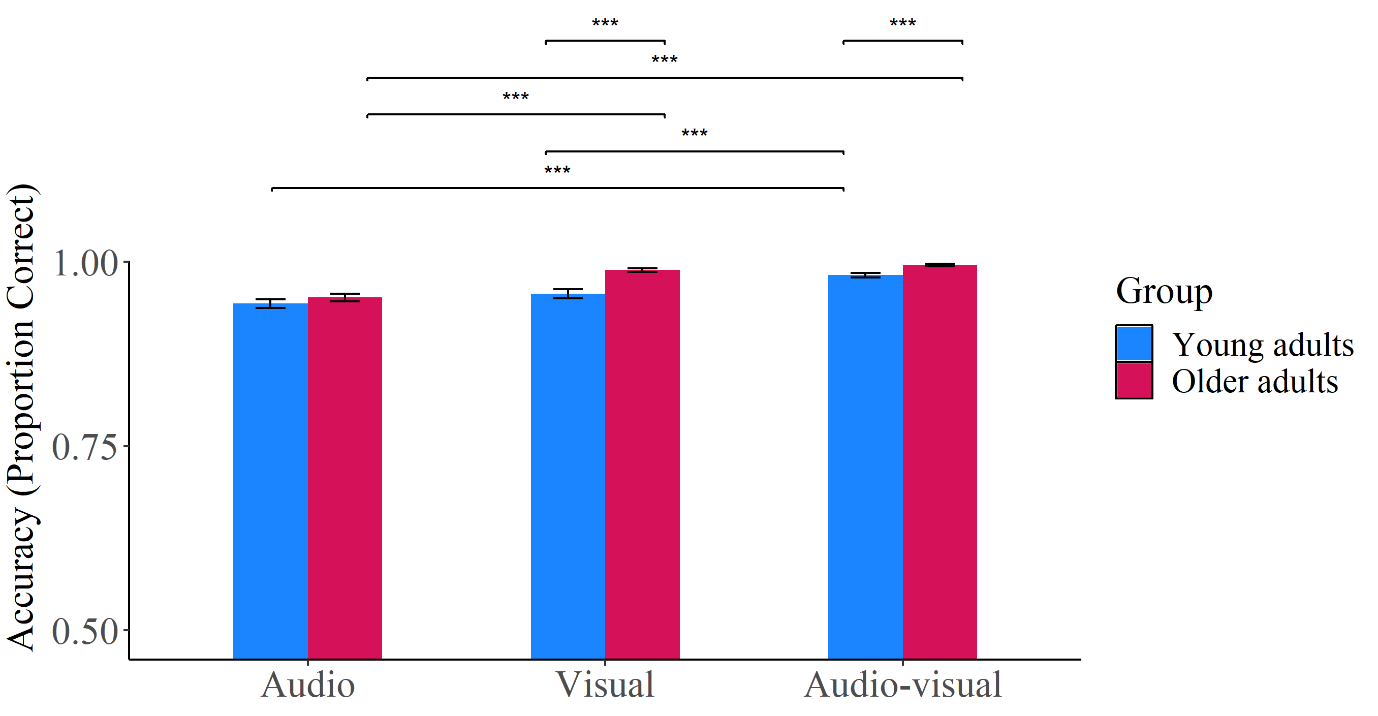


**Figure S1.** Lexical decision accuracy (proportion correct) for age (older and young) by modality (audio, visual and audio-visual). Error bars indicate standard error. * = <.05, ** = <.01, *** = <.001.


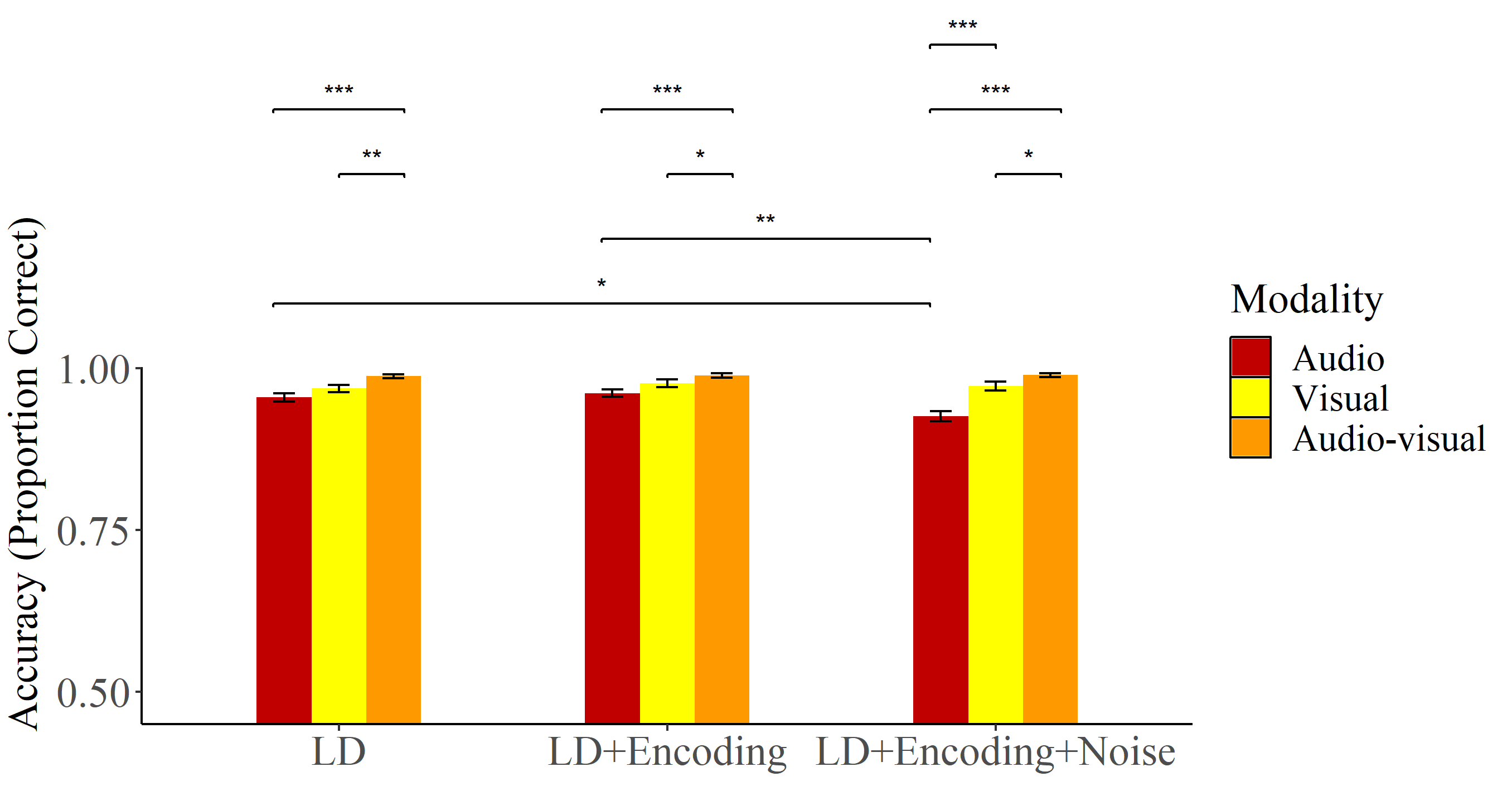


**Figure S2.** Accuracy (proportion correct) for modality (audio, visual and audio-visual) by load (LD only, LD + Encoding and LD + Encoding + noise). Error bars indicate standard error. * = <.05, ** = <.01, *** = <.001.

**Secondary analysis**

The secondary analysis will be presented below in accordance with the respective pre-registration (<https://doi.org/10.17605/OSF.IO/3QND6>). Greenhouse-Geisser correction was applied for non-sphericity. Effect sizes for ANOVAs are reported using partial eta squared. A 2 (Age: young, older) x 3 (Modality: audio, visual, audio-visual) x 3 (Load: encoding only, lexical decision + encoding, lexical decision + encoding + noise) x 12 (Serial position: 1–12) mixed ANOVA was conducted on the probability of correct recall. There was a main effect of age, *F*(1, 59) = 34.79, *p* < .001, η_p_^2^  = .37, with young participants recalling more words on average than older participants. There was a main effect of modality, *F*(2, 118) = 4.07, *p* = .020, η_p_^2^  = .07, with memory being superior when information was presented audio-visually compared to auditorily. There was a main effect of serial position, *F*(8.26, 487.21) = 20.06, *p* < .001, η_p_^2^  = .25, reflecting a strong primacy effect but no recency effects. There was no main effect of task, *F* <1. There was an interaction between serial position and age, *F*(11, 649) = 2.78, *p* = .002, η_p_^2^  = .05*,* indicating that young and older participants displayed similar primacy effects. There was an interaction between load and age, *F*(2, 118) = 7.27, *p* = .001, η_p_^2^  = .11, with memory being superior for young adults in the lexical decision and encoding task, whereas memory was superior for older adults in the encoding only task. There was no interaction between modality and load, *F*(4, 236) = 1.58, *p* = .181, η_p_^2^  = .03. There was no interaction between age, load and order, *F*(22, 1298) = 1.07, *p* = .369, η_p_^2^  = .02, and age, modality and load, *F*(4, 236) = 2.17, *p* = .073, η_p_^2^  = .04. All other interactions were non-significant, *F* < 1.

A 2 (Age: young, older) x 3 (Modality: audio, visual, audio-visual) x 3 (Load: encoding only, lexical decision + encoding, lexical decision + encoding + noise) mixed ANOVA was conducted on mean number of intrusions. There was a main effect of age, *F*(1, 60) = 34.79, *p* < .001, η_p_^2^  = .37, with young participants making significantly fewer intrusions than older participants. There was no main effect of load, *F*(2, 118) = 1.53, *p* = .219, η_p_^2^  = .03 and no main effect of modality, *F* < 1. There was no interaction between modality and load, *F*(4, 240) = 1.06, *p* = .376, η_p_^2^  = .02. All other interactions were non-significant, *F* < 1.

***Background Measures***

The Recall and lexical decision data were split by age (young and old) and Pearson’s correlations were conducted with the background measures: Digit Symbol Substitution (speed), multiple-choice part of the Mill Hill vocabulary test (crystallised intelligence) and the Speech in Noise test (hearing ability). After correcting for multiple tests, there were no systematic patterns in the data other than a significant negative relationship between processing speed and lexical decision response times in older adults (Supplementary Table S3).

**
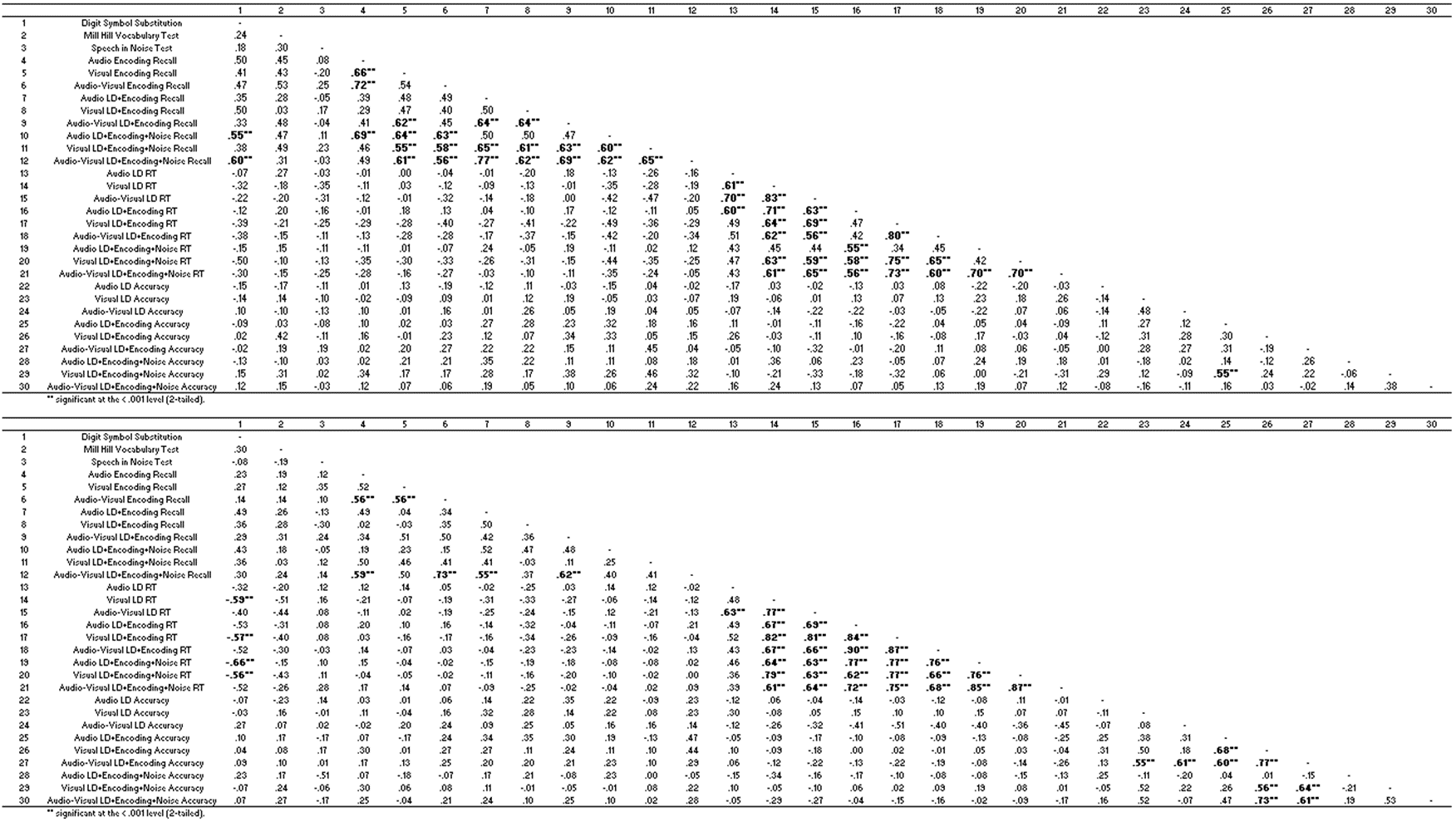
Table S3.** Top table: young adults. Bottom table: older adults. Both tables show correlations among the Digit Symbol Substitution test, Mill Hill vocabulary test, Speech in Noise test, modality (audio, visual audio-visual) and load (encoding, LD, LD+Encoding, LD+Encoding+Noise) on recall, log 10 response time, ms (RT) and accuracy.
